# Potential of *Bifidobacterium* *lactis* IDCC 4301 isolated from breast milk‐fed infant feces as a probiotic and functional ingredient

**DOI:** 10.1002/fsn3.3230

**Published:** 2023-02-08

**Authors:** O‐Hyun Ban, Won Yeong Bang, Hyeon Ji Jeon, Young Hoon Jung, Jungwoo Yang, Dong Hyun Kim

**Affiliations:** ^1^ Ildong Bioscience Gyeonggi‐do South Korea; ^2^ School of Food Science and Biotechnology Kyungpook National University Daegu South Korea

**Keywords:** antimicrobial, *Bifidobacterium* *lactis*, probiotics, safety assessment, whole‐genome sequence

## Abstract

Probiotics provide important health benefits to the host by improving intestinal microbial balance and have been widely consumed as dietary supplements. In this study, we investigated whether *Bifidobacterium* *lactis* IDCC 4301 (BL), isolated from feces of breast milk‐fed infants, is safe to consume. Based on the guidelines established by the European Food Safety Authority (EFSA), safety tests such as antibiotic susceptibility, hemolysis, toxic compound formation (i.e., biogenic amine and d‐lactate), single‐dose acute oral toxicity, and extracellular enzymatic activities were performed. In addition, toxigenic genes, antibiotic resistance genes, and mobile genetic elements were investigated by analyzing the genome sequence of BL. BL was susceptible to eight antibiotics except for vancomycin and the absence of transferable resistance in the genome of this strain implied that vancomycin resistance is likely to be intrinsic. With regard to phenotypic characteristics, there was no concern of toxicity of this strain. Furthermore, BL utilized various carbohydrates and their conjugates through the activity of various endogenous carbohydrate‐utilizing enzymes. Interestingly, the supernatant of the BL showed strong antipathogenic activity against various infectious pathogens. Therefore, we suggest that BL should be a safe probiotic and can be used as a functional ingredient in the food, cosmetic, and pharmaceutical industries.

## Graphical abstract

*Bifidobacterium* *lactis* IDCC 4301 was isolated from breast milk‐fed infant feces. Its potential was evaluated as a probiotic and functional ingredient. Its whole‐genome sequence was analyzed. Its safety was evaluated based on the guidelines suggested by FAO/WHO and EFSA. Its supernatant showed strong antimicrobial activity against various pathogens.

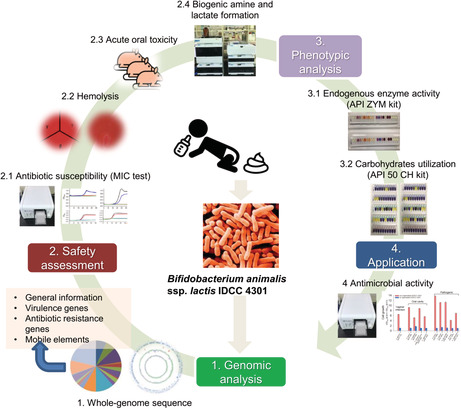

## 1 INTRODUCTION

Over the past decade, with increased emphasis on health, studies on the human microbiome have received more attention. The human microbiome refers to the collection of microbes in humans (Ursell et al., 2012). Because microbes living in humans reach up to 100 trillion cells, which corresponds to 10 times more than the number of human cells, and encodes 100 times more genes than the human genome, it is evident to say that humans are composed of microorganisms (Qin et al., 2010). Most microbes in humans live in the digestive system, particularly in the intestine, and their composition is closely linked to human health and disease (Backhed et al., 2005; Hall et al., 2017). The gut microbiota can change due to diet and lifestyle, resulting in dysbiosis, which causes many health disorders such as chronic and metabolic diseases (Conlon & Bird, 2014). Therefore, it is important to maintain intestinal microbial balance.

Probiotics are defined as live microorganisms that when administered in adequate amounts confer a health benefit to the host by improving the intestinal microbial balance by the Food and Agriculture Organization of United Nations (FAO) and World Health Organization (WHO) Expert Panel (FAO/WHO, 2002). Many studies have shown that probiotics have various health benefits, including amelioration of metabolic diseases such as diabetes and obesity (Kassaian et al., 2019), mental health problems including depression and anxiety (Abdrabou et al., 2018), cognitive disorder (Srivastav et al., 2019), and antimicrobial effects against pathogens (Stecher & Hardt, 2008). Therefore, probiotics are recognized as one of the best ways to manage health and have been commercially marketed as dietary supplements in various forms, which were authorized by the United States Food and Drug Administration (FDA) and European Food Safety Authority (EFSA).

Health benefits resulting from consumption of probiotics are strain specific (McFarland et al., 2018). Although there have been a variety of studies on the beneficial effects of probiotics, many researchers are trying to identify new probiotic strains with health benefits from various sources. To identify a new probiotic strain and use it as a dietary supplement, a safety assessment of the new probiotic strain is mandatory because probiotics are alive when administered, and there is no safety evidence regarding new strains. In addition, there have been several reports that adverse events, such as systemic infections, occur after the consumption of probiotics (Pradhan et al., 2020). Therefore, the FAO/WHO suggested that a safety assessment of new strains should be performed by following the guidelines for evaluating probiotics in food to prove that they are nonpathogenic and nontoxic to the host (FAO/WHO, 2002). According to these guidelines, common data such as whole‐genome sequencing and analysis to identify new strain, and minimal safety assessments such as antibiotic resistance, certain metabolic activities, side effects by oral administration in animal model, toxin production, and hemolytic activity should be required.

Among the probiotics, the most common genera are *Lactobacillus* and *Bifidobacterium*. In South Korea, 19 types of probiotic species are used as functional ingredients based on the standards and specifications of healthy functional foods announced by the Ministry of Food and Drug Safety (MFDS) of Korea, of which 12 species are *Lactobacilli* and 4 species are *Bifidobacteria* (Kim et al., 2020). The genus *Bifidobacterium* is a member of the dominant colonic microbiota, and it has various beneficial gastrointestinal effects: protection from colonic transit disorders and intestinal infections, prevention from colonic cancer, and improvement of immune system (Gill et al., 2001; Picard et al., 2005). Furthermore, it is nonpathogenic and is generally recognized as safe (GRAS) (Reuter, 2001). For these reasons, *Bifidobacterium* has been extensively used as probiotics over the past decade.

**FIGURE 1:**
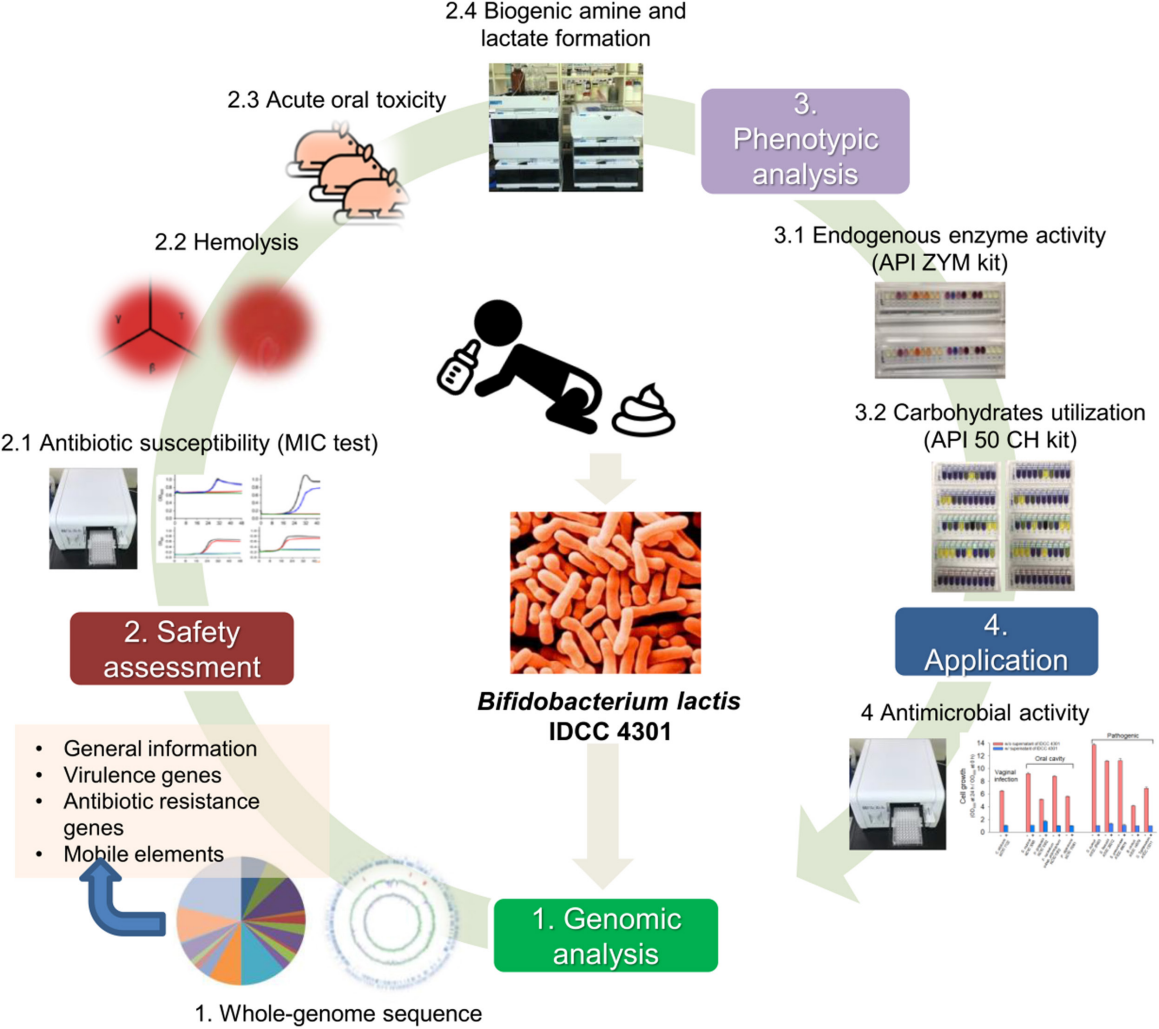
Overall research scheme in this study. Whole‐genome sequencing analysis, safety evaluation, phenotypic analysis of *Bifidobacterium lactis* IDCC 4301, and the potential of its application.

Particularly, *Bifidobacteria* play an important role in the intestine of infants. In breast milk‐fed infants, *Bifidobacteria* become dominant compared to formula‐fed infants, and they prevent various gastrointestinal diseases, including infant diarrhea caused by rotavirus infection (Harmsen et al., 2000; Picard et al., 2005). *Bifidobacteria* represent 80% of cultivable fecal bacteria in infants (Picard et al., 2005). Therefore, in this study, *Bifidobacterium* *lactis* IDCC 4301 (BL) was isolated from breast milk‐fed infant feces and its potential as a new probiotic strain was evaluated by performing genomic analysis, safety assessment, and antimicrobial activity tests for industrial application (Figure 1).

## 2 MATERIALS AND METHODS

### 2.1 Materials

De Man, Rogosa, and Sharpe (MRS), yeast malt (YM), brain–heart infusion (BHI) and nutrient media, and sheep blood agar plates were purchased from BD Difco. Tryptic Soy Broth (TSB) medium supplemented with hemin and menadione was purchased from KisanBio. HPLC‐grade acetonitrile was purchased from Sigma‐Aldrich.

### 2.2 Strain and culture conditions

The *Bifidobacterium lactis* IDCC 4301 (BL) strain (ATCC BAA‐2848) used in this study was isolated from the feces of breast milk‐fed infants and obtained from Ildong Bioscience. The strain was anaerobically cultured in MRS medium at 37°C with 0.5% CO_2_ in a static incubator for 16 h. For the antimicrobial activity tests of the supernatant from BL, three types of pathogenic microorganisms were tested. The first group comprised the vaginal infection‐related *Candida albicans* KCTC 7122. The second group included *Streptococcus mutans* KCTC 3065, *Porphyromonas gingivalis* KCTC 5352, *Fusobacterium nucleatum* subsp. *Polymorphum* KCTC 2488, and *Prevotella nigrescens* KCTC 15081, which are associated with the oral cavity. The third group included *Salmonella* Typhimurium ATCC 13311, *Bacillus cereus* ATCC 14579, *Staphylococcus aureus* ATCC 25923, *Enterococcus faecalis* ATCC 29212, and *Streptococcus pneumonia* ATCC 49619, which are associated with intestinal and respiratory infections. The cultivation conditions for each microorganism are listed in Table S1. In addition, various probiotic isolates were tested as controls, and the cultivation information is listed in Table S2.

### 2.3 Genomic analysis and bioinformatics

The genome of BL was sequenced and assembled using a PacBio RSII instrument with the Illumina platform (Macrogen). Antibiotic resistance genes were identified based on protein homologs using the ResFinder3.2 software and compared to the CARD database (https://card.mcmaster.ca/), and the determination of resistance genes was confirmed according to the CARD criteria (search parameters for sequence identity >80% and coverage >60%). To analyze virulence genes, genomic sequence similarities to toxigenic genes with the BLASTn algorithm were evaluated using the Virulence Factor Database (http://www.mgc.ac.cn/VFs/; thresholds for the identification were identity >70% and coverage >70%). In addition, mobile genetic elements were searched using the BLASTp algorithm (for transposases and plasmids) and the PHASTER web‐based program (for prophage regions).

### 2.4 Safety assessments

#### 2.4.1 Antibiotic susceptibility test

The antibiotic sensitivity of BL was investigated based on the EFSA recommendations (Kim et al., 2021; Rychen et al., 2018). Nine antibiotics (ampicillin, vancomycin, gentamicin, kanamycin, streptomycin, erythromycin, clindamycin, tetracycline, and chloramphenicol), which are typically used to treat enterococcal infections, were tested. A 150 μL of aliquot from BL culture, grown overnight was transferred to 15 mL of fresh MRS broth. When the cell density reached 1 × 10^6^ CFU/mL, 50 μL of the cell culture was transferred to a 96‐well plate containing 50 μL of the antibiotic solution at different concentrations. The final concentrations of cells and antibiotics were 3.3–6.6 × 10^5^ CFU/mL and 0.125–1024 μg/mL, respectively. The plate was then incubated at 37°C, and the optical density (OD) was measured at 600 nm using a microplate reader (BioTek) for 20 h. Finally, the minimum inhibitory concentration (MIC) was determined as the lowest concentration that completely inhibited the growth of BL.

#### 2.4.2 Hemolysis test

The *β*‐hemolytic activity of BL was tested according to the guidelines of American Society for Microbiology (Buxton, 2005). BL was streaked as a “T” in the upper right one‐third of the sheep blood agar plate, *Staphylococcus aureus* subsp. *aureus* ATCC 25923 (positive control) was streaked “β” in the bottom one‐third of the plate, and *Lactobacillus reuteri* IDCC 3701 (*γ*‐control) was streaked “*γ”* in the upper left one‐third of the plate. Hemolytic activity was determined by clear zones around the colonies.

#### 2.4.3 Biogenic amine production

The ability of BL to produce biogenic amines, such as tyramine, histamine, putrescine, 2‐phenethylamine, and cadaverine, was investigated. The supernatant of BL grown in MRS medium was obtained by centrifugation at 1842 x*g* and 4°C for 30 min and filtered through a 0.22 μm filter. Derivatization was then performed to quantify biogenic amines in the supernatant using HPLC. First, 0.75 mL of the supernatant was mixed with 0.75 mL of 0.1 M HCl and filtered through a 0.45 μm filter to extract biogenic amines. Then, 1 mL of the filtered sample was incubated in a water bath at 70°C for 10 min, followed by the addition of 200 μL of saturated NaHCO_3_, 20 μL of 2 M NaOH, and 0.5 mL of dansyl chloride (10 mg/mL in acetone). The derivatized sample was mixed with 200 μL of proline (100 mg/mL in H_2_O) and incubated in the dark at 25°C for 15 min. The volume of the mixture was made up to 5 mL using acetonitrile (HPLC grade). The biogenic amines in the mixture were quantified by HPLC (LC‐NETII/ADC; Jasco) equipped with an Athena C18 column (4.6 × 250 mm; ANPEL Laboratory Technologies) using an aqueous acetonitrile solution (67:33 of H_2_O, v/v) as the mobile phase at a flow rate of 0.8 mL/min (Ban et al., 2020).

#### 2.4.4 Single‐dose acute oral toxicity of *B. lactis* IDCC 4301

This test was conducted by the Korea Testing and Research Institute in accordance with Organization for Economic Co‐operation and Development (OECD) guidelines for testing chemicals and acute oral toxicity (OECD, 2002). The protocol for this test is illustrated in Figure 4a. This test was conducted on Crl:CD (Sprague–Dawley) female rats. The freeze‐dried BL powder, corresponding to 3.2 × 10^11^ cells in 10‐mL sterile distilled water used as an injection vehicle, was administered only once orally at a dose of 300 mg/kg body weight (BW) (first and second steps) and 2000 mg/kg BW (third and fourth steps). Three animals were used in each step. Mortality, clinical signs, BW, and necropsy findings were recorded over 14 days.

### 2.5 Metabolic characteristics

#### 2.5.1 Lactate formation

To determine the ratio of l‐ to d‐lactate produced by BL, cells were incubated in MRS medium at 37°C for 24 h. The supernatant was obtained by centrifugation and filtered through a 0.22 μm pore size membrane. l‐ and d‐lactate were quantified using an assay kit (Megazyme) according to the manufacturer's protocol. The absorbance of the reactants was measured at 340 nm using a microplate reader (Bio‐Rad).

#### 2.5.2 Enzymatic activity test

The enzymatic activities of BL were investigated using an API ZYM kit (bioMérieux), which can test 19 different types of enzymes according to the manufacturer's protocol. BL was grown on MRS broth for 16 h and then harvested by centrifugation. The harvested cells were resuspended in phosphate‐buffered saline, resulting in cell density of 1.8 × 10^9^ CFU/mL. The suspension was then added to cupules containing each substrate and then incubated for 4 h at 37°C. After incubation, one droplet of ZYM reagents A and B was added, and color changes in each cupule were observed.

#### 2.5.3 Ability to utilize various carbohydrates

The ability of BL to utilize various carbohydrates was investigated using an API 50 CHL/CHB kit (bioMérieux) containing 49 different types of carbohydrates, according to the manufacturer's protocol. BL was grown on an MRS broth for 16 h and then harvested by centrifugation at 4146 x*g* and 4°C for 10 min. The harvested cells were suspended in 10‐mL API 50 CHL/CHB medium. The cell density was adjusted to 6.0 × 10^8^ CFU/mL and then added into each cupule containing each carbohydrate. After incubation for 48 h at 37°C, color changes in each cupule were observed.

### 2.6 Antimicrobial activity against various pathogenic microorganisms

BL was incubated in MRS broth at 37°C for 16 h anaerobically. The supernatant was obtained by centrifugation at 4146 x*g* and 4°C for 10 min, filtered through a 0.45 μm membrane, and used to test for antimicrobial activity. One hundred microliters of the filtered supernatant was added to a 96‐well plate with 100 μL of each pathogen, whose cell density was 1.5 × 10^6^ CFU/mL, and the plate was incubated in appropriate culture conditions (Table S1). Antimicrobial activity was evaluated by measuring the OD at 600 nm using a microplate reader (BioTek) to compare the cell growth with the supernatant to that without control, which was expressed as OD_600_ at 24 h divided by OD_600_ at 0 h.

## 3 RESULTS AND DISCUSSION

### 3.1 Genomic analysis of the *B. lactis* IDCC 4301

**FIGURE 2:**
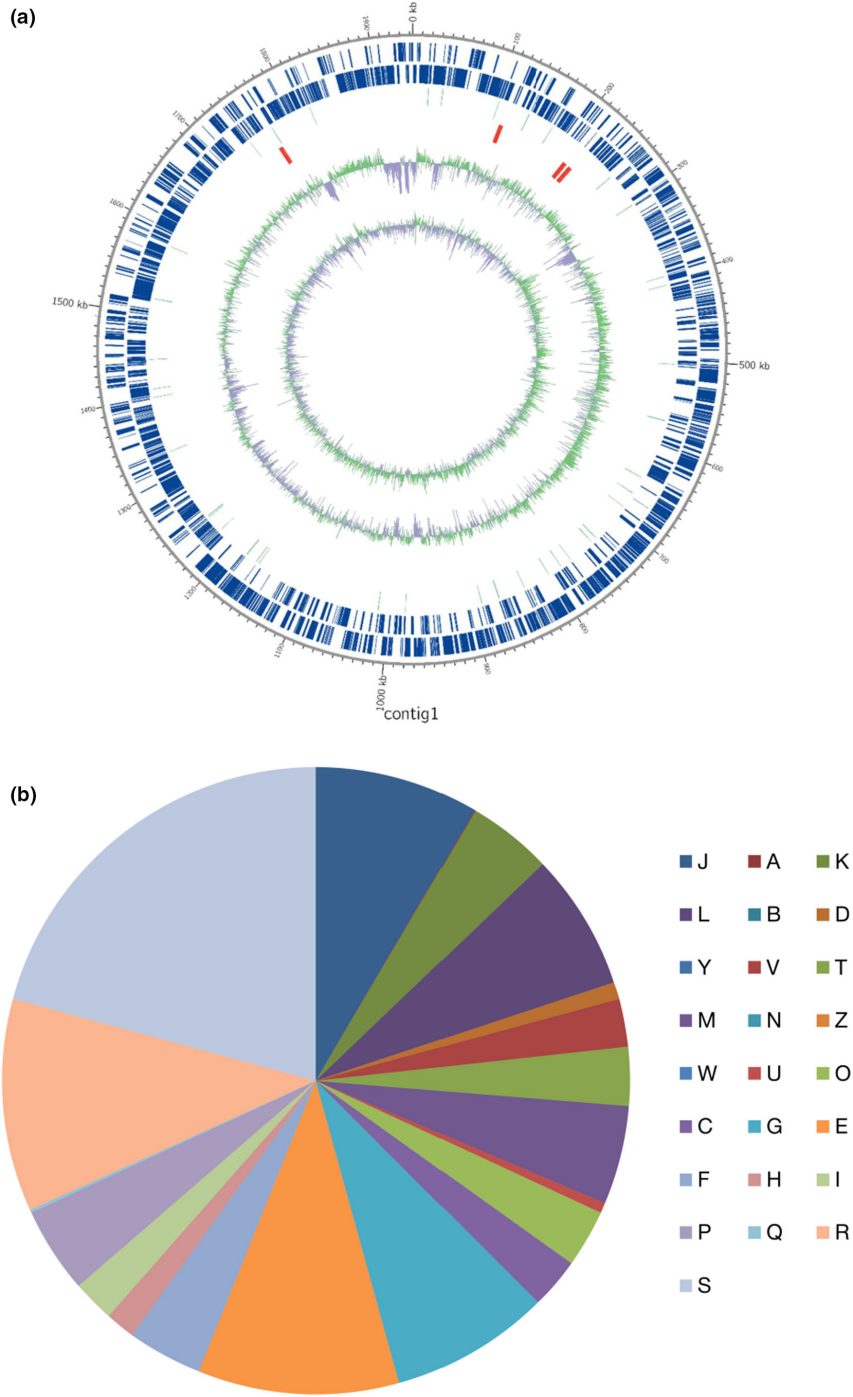
Whole‐genome information of *Bifidobacterium lactis* IDCC 4301 (BL). (a) A circular map of BL was obtained using contig 1 annotation results. Marked characteristics are shown from the outside to the center; coding DNA sequence (CDS) on the forward strand, CDS on the reverse strand, tRNA, rRNA, GC content, and GC skew. (b) Functional genes in BL were annotated using the Eggnog database. The detailed description, count, and ratio of the Eggnog annotation are listed in Table S3.

To accept a probiotic as a functional ingredient, a complete genome sequence of a target microorganism should be analyzed to identify the phenotype and genotype of the strain. It is important to identify the genus and species of probiotic strain because probiotic effects result from strain‐specific probiotic (Campana et al., 2017; Gaisawat et al., 2021; Kekkonen et al., 2008). A total of 19 types of probiotic species, which consist of five genera (*Lactobacillus, Lactococcus, Streptococcus, Bifidobacterium*, and *Enterococcus*), have been approved as probiotics by the MFDS of Korea, based on WHO/FAO guidelines (Kim et al., 2020). In addition, by analyzing the genome of the target probiotic strain to determine whether this strain contains toxigenic and antibiotic resistance genes and whether these genes are transferable to other commensal microbes, valuable information regarding its safety can be obtained. As a result of genomic analysis (Table 1 and Figure 2a), the bacterium isolated from the feces of breast‐milk‐fed infants showed the highest similarity to the type strain of *Bifidobacterium* *lactis* UR1 based on an average nucleotide identity (ANI) of 99.95%. The genome size was approximately 1.94 Mb with a GC content of 60.48%. In total, 1813 functional genes were predicted and grouped using the EggNOG database (Figure 2b). Interestingly, among the functional categories of genes, those related to amino acid transport and metabolism (E, 10.36%) and carbohydrate transport and metabolism (G, 8.21%) belonged to the first and third of the five major functional categories, respectively, except for genes indicating unknown and general functions (Figure 2b). Thus, it is suggested that this strain has the potential to help digestion and absorption of various nutrients, including amino acids and carbohydrates in the host.

**TABLE 1:** Genomic information of *Bifidobacterium* *lactis* IDCC 4301.

Identification	*B. lactis*
ANI value (%)	99.95
No. of 16S rRNA genes	4
Genome size (bp)	1,942,819
GC contents (%)	60.48
CDS	1813

Abbreviations: ANI, average nucleotide identity; CDS, coding DNA sequence.

In terms of safety of *Bifidobacterium lactis* IDCC 4301 (BL), virulence genes, antibiotic resistance genes, and mobile genetic elements (such as transposases, prophage regions, and conjugal transfer plasmids) were also predicted. There were no virulence genes in this genome, based on the BLASTn algorithm using VFDB. In terms of antibiotic resistance genes, among the nine antibiotic resistance genes (ampicillin, vancomycin, gentamycin, kanamycin, streptomycin, erythromycin, clindamycin, tetracycline, and chloramphenicol) the tetracycline resistance gene, *tetW*, was detected on the genome of the BL (Table 2). *Bifidobacterium* spp. showed a high prevalence and wide distribution of resistance to tetracycline (Aires et al., 2007; Ammor et al., 2008). For example, it was found that 33% of *Bifidobacteria* showed tetracycline resistance and 83% of the tetracycline‐resistant isolates carried the *tetW* gene (Aires et al., 2007). Furthermore, all strains of *B.* *lactis* described to date show medium‐level resistance to tetracycline (Gueimonde et al., 2010).

**TABLE 2:** Antibiotic resistance gene in *Bifidobacterium* *lactis* IDCC 4301.

Resistance gene	Identity (%)	Size (bp)	Location	Accession number
tet(W)	98.85	1914	1,386,827–1,388,739	FN396364

There was no transferability of *tetW* based on the analysis of mobile genetic elements (Tables S3 and S4). Transposase binds to the end of a transposon and catalyzes its movement to another part of the genome, resulting in the promotion of gene transfer between microorganisms (Bennett, 2008). In this strain, eight genes encoding transposases were identified, but there was no transposase in the 10‐kb regions surrounding the *tetW* gene, which was located at the 1,386,827–1,388,739 bp (Table S4). Thus, *tetW* gene transfer by transposases is not possible. In addition, the prophage regions in this strain were located at 1,653,553–1,661,349 bp, and this position was not related to the *tetW* gene (Table S5). Bacteriophages typically act as important agents for horizontal gene transfer (Canchaya et al., 2003). Bacterial conjugation is another mechanism of horizontal gene transfer that is mediated by plasmids (Sørensen et al., 2005). Plasmids or other extra replicons were not present in this strain. In accordance with our analysis, it was reported that *bifidobacteria* rarely harbor plasmids (Wang et al., 2017). Consequently, the probability of the *tetW* gene transfer to other microbes is extremely low.

### 3.2 Antibiotic susceptibility of the *B. lactis* IDCC 4301

It is essential to investigate the antibiotic resistance of the target strain because there is a possibility of resistance transfer from probiotics to commensal microbes or pathogens in humans, which may result in severe consequences, especially in immunocompromised patients during clinical surgery (Sanders et al., 2010). Whereas most microorganisms can survive at low concentrations of many antibiotics, resistance is defined as the capacity to grow at antibiotic concentrations similar to those reached in the human body during therapeutic intervention (Mathur & Singh, 2005). According to genomic analysis for antibiotic resistance genes, BL harbors a tetracycline resistance gene, *tetW*. Therefore, the antibiotic sensitivity of this strain was investigated by determining the MICs of antibiotics including tetracycline (Table 3). As the strain did not show phenotypic resistance to tetracycline over the EFSA breakpoint (8 μg/mL), resistance due to this gene is not considered of large concern. According to the previous studies on *Bifidobacterium* spp., it was reported that *B.* *lactis* is widely known to harbor the *tetW* gene, and there is no possibility of *tetW* gene transfer to other microorganisms (FDA, 2019a; Gueimonde et al., 2010; Ku et al., 2020). However, unlike our results, many of the strains listed in other studies were resistant to tetracycline, with MICs over the cut‐off value (8 μg/mL) (FDA, 2019a, 2019b; Kim et al., 2018; Ku et al., 2020). Nevertheless, the FDA has recognized these strains as GRAS and to be used as probiotics. Furthermore, BL was susceptible to all the antibiotics tested in this study, except for vancomycin. While this strain was found to have phenotypic antibiotic resistance to vancomycin, it was not found to contain antibiotic resistance genes in the genomic sequence of this antibiotic. The absence of genetic resistance in the genome of this strain implies that its resistance is likely to be intrinsic and not likely to be horizontally transferrable to other bacteria (Kheadr et al., 2007). Finally, we conclude that the BL strain is safe in terms of antibiotic resistance.

**TABLE 3:** MICs of *Bifidobacterium lactis* IDCC 4301 against various antibiotics.

	Ampicillin	Vancomycin	Gentamicin	Kanamycin	Streptomycin	Erythromycin	Clindamycin	Tetracycline	Chloramphenicol
Cut‐off value (μg/mL)^a^	2	2	64	Not required	128	1	1	8	4
*B. lactis* IDCC 4301	<0.125/S^b^	>512/R^c^	16–32/S	64–256	64–128/S	0.125–0.5/S	0.125–0.5/S	8/S	1–2/S

^a^ These cut‐off values are based on European Food Safety Authority recommendations (Rychen et al., 2018).

^b^ This indicates that IDCC 4301 is susceptible to the antibiotic.

^c^ This indicates that *Bifidobacterium lactis* IDCC 4301 is resistant to the antibiotic.

### 3.3 Hemolytic activity of the *B. lactis* IDCC 4301

**FIGURE 3:**
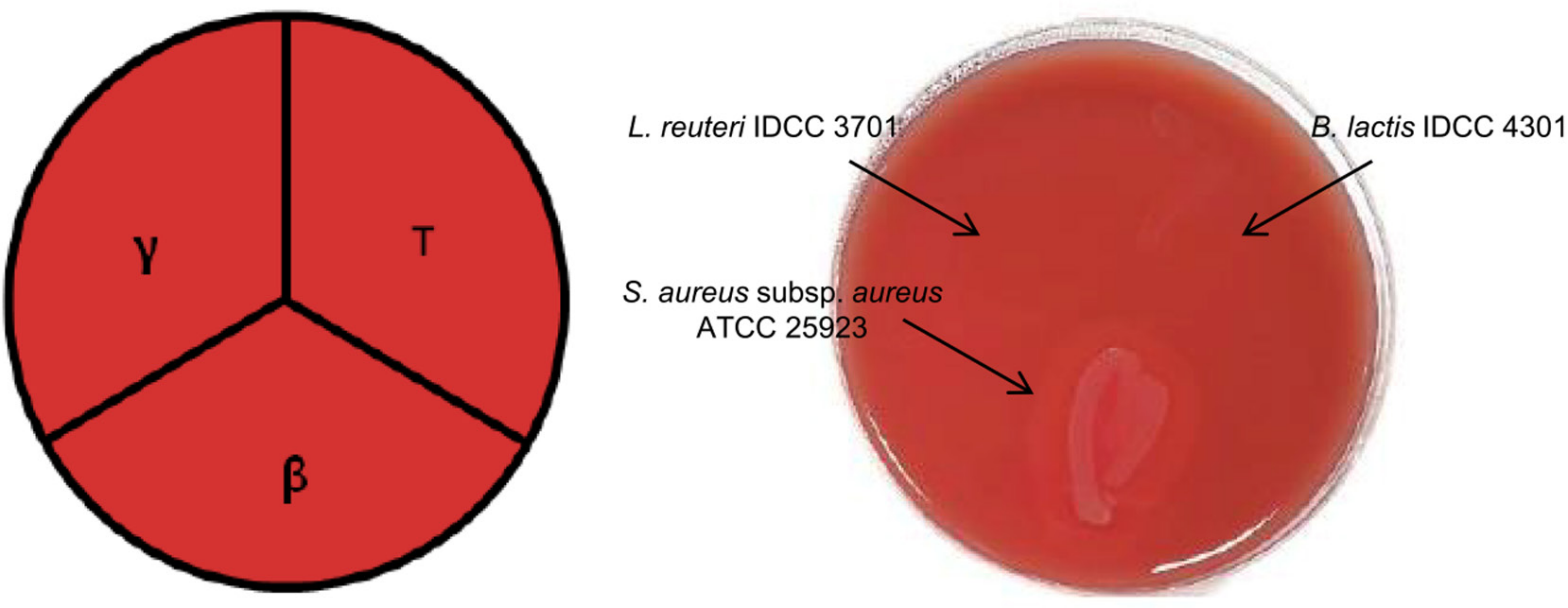
Hemolytic activities of *Bifidobacterium lactis* IDCC 4301. *Staphylococcus aureus* subsp. *aureus* ATCC 25923 and *Lactobacillus reuteri* IDCC 3701 were used as the *β*‐control (positive) and *γ*‐control (negative), respectively.

Hemolysis is the ability to lyse red blood cells by disrupting the membrane and pathogens are mostly hemolytic to invade the human body (Kang et al., 2019). Thus, it is essential to test whether the target strain used as a probiotic shows hemolytic activity. The FAO/WHO Working Group Guidelines for the Evaluation of Probiotics in Food recommends a hemolysis test to ensure probiotic safety (FAO/WHO, 2002). Depending on the characteristics of the zone produced by the tested bacterium, it can be distinguished as *α*‐hemolysis (partial lysis showing a deep green zone), *β*‐hemolysis (complete lysis showing a clear zone), and *γ*‐hemolysis (no lysis resulting in no zone). Among probiotics, *Lactobacillus* spp. is known to have *α*‐hemolytic activity as well as strong *β*‐hemolytic activities (Goldstein et al., 2015; Gómez et al., 2016). In this study, BL showed *γ*‐hemolysis because there was no clear zone on the blood agar plate (Figure 3). This result was consistent with the whole‐genome sequencing results, which showed that there were no virulence genes (i.e., hemolysin) in this strain.

### 3.4 Production of biogenic amines and d‐lactate by the *B. lactis* IDCC 4301

Recently, cases of adverse events such as headache, diarrhea, and flushing after probiotic administration have been reported. Biogenic amines are basic nitrogenous compounds with one or more amine groups, produced by the decarboxylation of amino acids or by the amination and transamination of aldehydes and ketones (Santos, 1996). Biogenic amines are known to cause various adverse physiological effects on human health, including hypertension, headache, vomiting, diarrhea, and fatal outcomes (Santos, 1996). Biogenic amines are present in various fermented foods including seafood (Doeun et al., 2017). For example, fish containing high levels of histidine, such as tuna, mackerel, anchovy, herring, and amberjack, are spoiled by bacteria, and histidine in fish is converted to histamine, causing adverse symptoms (Kim et al., 2013). Our results showed that BL did not produce biogenic amines such as tyramine, histamine, putrescine, 2‐phenethylamine, or cadaverine (data not shown).

Lactate is widely produced during food processing, particularly in foods fermented by microorganisms. Lactic acid, together with short‐chain fatty acids such as acetic, propionic, and butyric acids, make valuable contributions to human colon health by maintaining a low pH environment, resulting in the prevention of pathogen survival (Aldunate et al., 2015). In humans, l‐lactate is metabolized, whereas d‐lactate is accumulated due to difficulty to be metabolized. Several pathways are involved in d‐lactate production. Probiotics contain dl‐lactate racemase, which converts l‐lactate to d‐lactate. d‐lactate accumulation may only occur in cases of impaired d‐lactate metabolism and/or in subjects with disturbed gastrointestinal function following bowel resection or short bowel syndrome (Pohanka, 2020). Therefore, FAO/WHO guidelines require information on dl‐lactate formation by probiotics (FAO/WHO, 2002). In this study, lactate production by BL was evaluated (Table 4). BL produced more l‐lactate than d‐lactate: 17.37 mg/mL of l‐lactate (82.87%) and 3.59 mg/mL of d‐lactate (17.13%). The value of d‐lactate formation and the ratio of d‐ to l‐lactate were much lower than those of other lactic acid bacteria such as *L. plantarum*, *L. acidophilus*, *L. reuteri*, and *L. delbrueckii* (Bang et al., 2021; Lee et al., 2021; Sulemankhil et al., 2012). In particular, *L. reuteri* NCIMB 30253 and *L. delbrueckii* ATCC 11842 produced higher ratios of d‐lactate than l‐lactate, with 6:5 and 12:11 ratios of d‐ to l‐lactate, respectively (Sulemankhil et al., 2012). Finally, we conclude that the BL strain is safe in terms of nontoxic compound formation.

**TABLE 4:** The ratio of l‐ to d‐lactate produced by *Bifidobacterium lactis* IDCC 4301.

Strain	l‐lactate (mg/mL)	d‐lactate (mg/mL)	Ratio (%)	
			l‐form	d‐form
*B. lactis* IDCC 4301	17.37 ± 0.23	3.59 ± 0.03	82.87	17.13

### 3.5 Single‐dose acute oral toxicity of the *B. lactis* IDCC 4301

**FIGURE 4:**
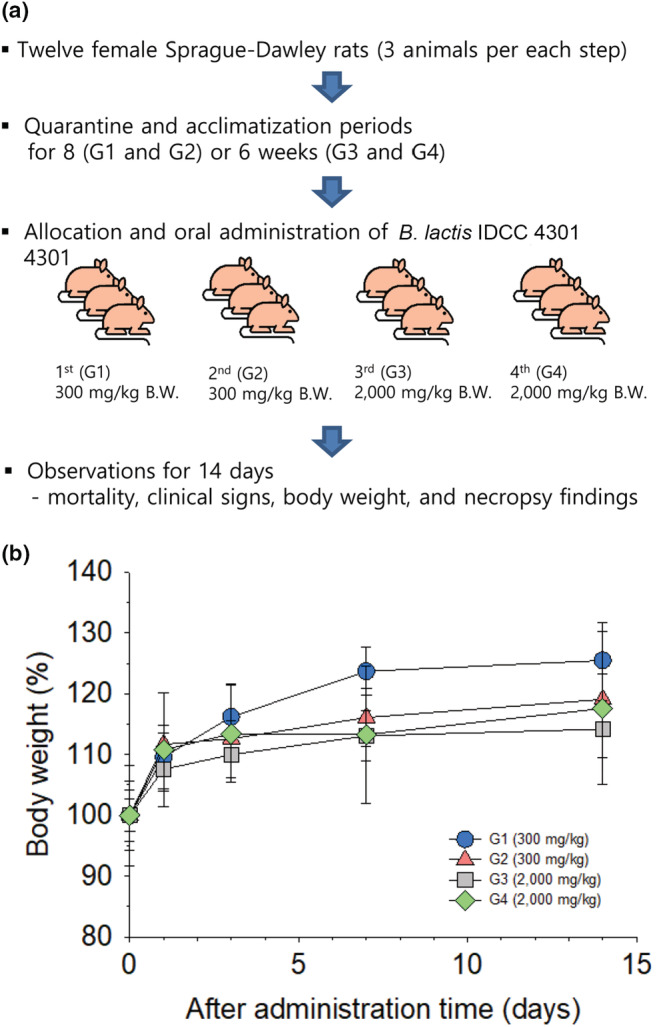
Single‐dose acute oral toxicity test of *Bifidobacterium lactis* IDCC 4301. (a) Schematic diagram of the test procedure. (b) Percentage change of body weight.

Probiotics are a class of living microorganisms. Thus, although most *Bifidobacterium* species are generally recognized as GRAS, the oral administration toxicity test of the new strain as a probiotic is a prerequisite. Therefore, following the OECD test guideline for testing chemicals in section 4, Test No. 423 “Acute Oral Toxicity‐Acute Toxic Class Method” (OECD, 2002), the single‐dose acute oral toxicity of BL was tested in SD rats. The BL administration procedure is shown in Figure 4a. Mortality, clinical signs, body weight (BW), and necropsy findings were recorded for 14 days after oral administration. No test substance‐related mortality or clinical signs were observed during the study period (Table S6). With regard to BW changes, although a 0.3%–2.3% BW decrease was observed in some individuals, given the overall tendency of weight gain, it was assumed to be accidental weight changes in the subjects (Figure 4b). For necropsy, there were no abnormal findings caused by the administration of the test substance. Therefore, BL was classified into the Globally Harmonized Classification System for Chemical Substances and Mixtures Category 5 or was not classified in this study (Winder et al., 2005), suggesting that BL is not toxic to the host.

### 3.6 Endogenous enzymatic activity and carbohydrate fermentation profile of *B. lactis* IDCC 4301

It is important to investigate the endogenous enzymatic activities of newly isolated strain to evaluate its safety and utilization of nutrients in the gut environment. In this study, 19 types of enzymatic activities, including diverse esterases, lipases, peptidases, and glycosidases, were investigated in the BL strain using the API ZYM kit (Table 5). BL showed esterase, lipase, leucine arylamidase, cystine arylamidase, naphthol‐AS‐BI‐phosphohydrolase, *α*‐galactosidase, *β*‐galactosidase, *α*‐glucosidase, and *β*‐glucosidase activities (Table 5). With regard to safety, probiotics should not show *β*‐glucuronidase activity because this enzyme is related to the formation of carcinogenic compounds, including cyclins, which are associated with the risk of developing colorectal cancer (Kim & Jin, 2001). In this study, BL strain did not exhibit *β*‐glucuronidase activity (Table 5).

**TABLE 5:** Endogenous enzymatic activities of *Bifidobacterium lactis* IDCC 4301.

No.	Enzyme	Result	No.	Enzyme	Result
1	Alkaline phosphatase	−^a^	11	Naphthol‐AS‐BI‐phosphohydrolase	+
2	Esterase	+^b^	12	*α*‐Galactosidase	+
3	Esterase lipase	+	13	*β*‐Galactosidase	+
4	Lipase	−	14	*β*‐Glucuronidase	−
5	Leucine arylamidase	+	15	*α*‐Glucosidase	+
6	Valine arylamidase	+^W^ ^c^	16	*β*‐Glucosidase	+
7	Cystine arylamidase	+	17	N‐acetyl‐*β*‐glucosaminidase	−
8	Trypsin	−	18	*α*‐Mannosidase	−
9	*α*‐Chymotrypsin	−	19	*α*‐Fucosidase	−
10	Acid phosphatase	+			

^a^ −: No activity.

^b^ +: Activity.

^c^ +^W^: Weak activity.

Furthermore, probiotics utilize various carbohydrates in the form of dietary fiber (called prebiotics) as nutrients and produce beneficial secondary metabolites for human colon cells, resulting in a healthier digestive system (Markowiak & Śliżewska, 2017). BL showed *α*‐galactosidase, *β*‐galactosidase, *α*‐glucosidase, and *β*‐glucosidase activities, which can digest conjugates of glucose and galactose from various foods (Table 5). Additionally, the ability of BL to utilize 49 types of carbohydrates was investigated using the API 50 CH kit (Table 6). BL utilized most of the common hexoses (ribose, galactose, d‐glucose, d‐fructose, and d‐mannose) and pentose sugars (l‐arabinose and d‐xylose), which are widely distributed in nature. In addition, this strain can metabolize various disaccharides, including maltose, lactose, melibiose, sucrose, trehalose, and gentiobiose. Interestingly, this strain utilizes plant‐based glucoside derivatives, including amygdalin and esculin. These types of plant‐based glycosides are used as phytochemicals, and their bioactivity and bioavailability can be modified into acylglycones by glycoside hydrolases, particularly *β*‐glucosidase of gut microorganisms (Modrackova et al., 2020). The *β*‐glucosidase activity of *Bifidobacterium* is species specific and increases the bioactivity of plant‐based glycosides (Modrackova et al., 2020). In this study, BL showed *β*‐glucosidase activity and utilized amygdalin and esculin (Tables 5 and 6, respectively). Therefore, BL has great potential for improving the intestinal environment because it can utilize various carbohydrate sources and produce bioactive compounds by utilizing plant‐based glycosides via its *β*‐glucosidase activity.

**TABLE 6:** Carbohydrate utilization by *Bifidobacterium lactis* IDCC 4301.

No.	Substrate	Result	No.	Substrate	Result	No.	Substrate	Result
1	Glycerol	−^a^	18	Mannitol	−	35	d‐Raffinose	+
2	Erythritol	−	19	Sorbitol	−	36	Amidon	−
3	d‐Arabinose	−	20	*α*‐Methyl‐d‐mannoside	−	37	Glycogen	−
4	l‐Arabinose	+^W^ ^b^	21	*α*‐Methyl‐d‐glucoside	+^W^	38	Xylitol	−
5	Ribose	+^c^	22	N‐Acetyl‐glucosamine	−	39	Gentiobiose	+ ^W^
6	d‐Xylose	+^W^	23	Amygdalin	+	40	d‐Turanose	−
7	l‐Xylose	−	24	Arbutin	−	41	d‐Lyxose	−
8	Adonitol	−	25	Esculin	+	42	d‐Tagatose	−
9	*β*‐Methyl‐xylose	−	26	Salicin	−	43	d‐Fucose	−
10	Galactose	+	27	Cellobiose	−	44	l‐Fucose	−
11	d‐Glucose	+	28	Maltose	+	45	d‐Arabitol	−
12	d‐Fructose	+^W^	29	Lactose	+	46	l‐Arabitol	−
13	d‐Mannose	+^W^	30	Melibiose	+	47	Gluconate	−
14	l‐Sorbose	−	31	Sucrose	+	48	2‐Keto‐gluconate	−
15	Rhamnose	−	32	Trehalose	+	49	5‐Keto‐gluconate	−
16	Dulcitol	−	33	Inulin	−			
17	Inositol	−	34	Melezitose	−			

^a^ −: No utilization.

^b^ +^W^: Weak utilization.

^c^ +: Utilization.

### 3.7 Antimicrobial effect of the *B. lactis* IDCC 4301 on pathogenic microorganisms

**FIGURE 5:**
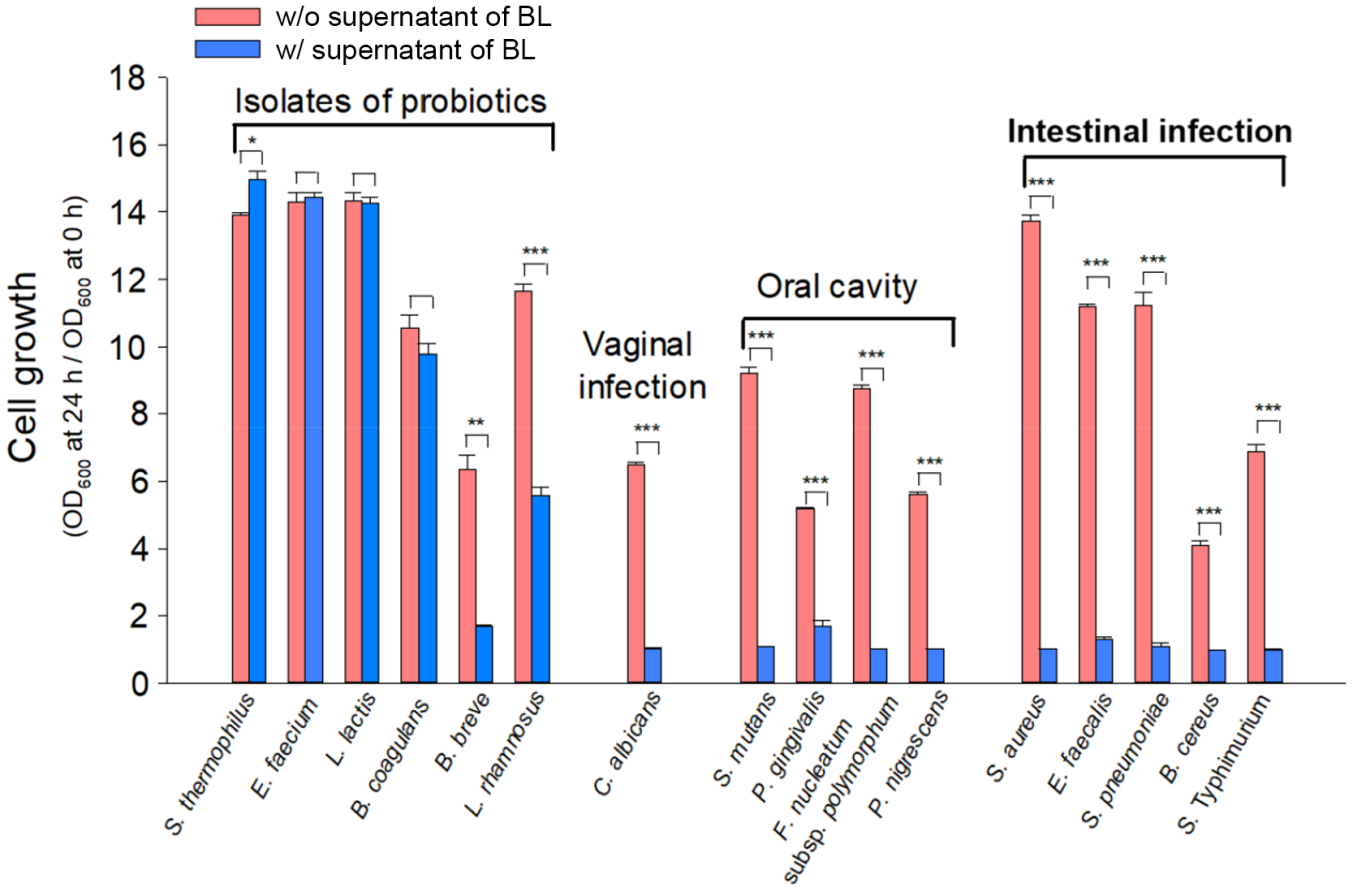
Antimicrobial effects of the supernatant from *Bifidobacterium lactis* IDCC 4301 on various pathogenic microorganisms. Antimicrobial activity was indicated as cell growth; OD_600_ at 24 h divided by OD_600_ at 0 h. *p < 0.05, **p < 0.01, ***p < 0.001.

There are a lot of evidences regarding the various health benefits of beneficial gut bacteria. By changing the gut microbiota, the bacteria provide competitive adherence to the mucosa and epithelium against pathogens, thus improving gut barrier function and boosting immune system (Plaza‐Diaz et al., 2019). Recently, the term postbiotics have also emerged, and the concept does not require the viability of desirable gut bacteria for health benefits to the hosts (Salminen et al., 2021). For example, four different fractions from *Bifidobacterium bifidum* BGN4 such as lysed whole‐cell, cell‐free extracts, purified cell wall, and culture supernatant showed different beneficial effects on immune reactions (Lee et al., 2002). In addition, several studies have proven that probiotics exhibited various bioactivities including immunomodulatory, antiinflammatory, antioxidant, antihypertensive, hypocholesterolemic, and antimicrobial effects (Aguilar‐Toalá et al., 2018). Therefore, in this study, the potential of BL as a postbiotic was evaluated by testing antimicrobial effects against various types of pathogens using its culture supernatant (Figure 5). The microorganisms tested in this study belonged to three categories: vaginitis infection, such as *C. albicans*; oral health, such as *S. mutans*, *P. gingivalis*, *F. nucleatum* subsp. *polymorphum*, and *P. nigrescens*; and infectious pathogens in the intestine, such as *S. aureus*, *E. faecalis*, *S. pneumonia*, *B. cereus*, and *S*. Typhimurium (Table S1). We also tested the inhibitory effect of the BL supernatant on other probiotic isolates such as *Streptococcus thermophilus*, *Enterococcus faecium*, *Lactococcus lactis*, *Bacillus coagulans, Bifidobacterium breve*, and *Lactobacillus rhamnosus* as controls; the BL strain should not inhibit the growth of beneficial microorganisms. Interestingly, the BL supernatant strongly inhibited the growth of all the tested pathogens (Figure 5). The presence of metabolites such as short‐chain fatty acids (SCFAs) (Ibrahim & Bezkorovainy, 1993), lipophilic molecules (Lievin et al., 2000), and bacteriocin (bifidocin B) (Yildirim & Johnson, 1998) in the supernatant may explain its antimicrobial activity. In this study, we found that BL produces lactic and acetic acids at 2.2 and 2.5 g/L, respectively. In addition, organic acids from cell‐free supernatants of *L. reuteri* KCTC strains show antimicrobial activities against acne‐related bacteria, such as *Propionibacterium acnes* and *S. epidermidis* (Kang et al., 2012). In this study, the supernatant of BL showed an inhibitory effect on *S. aureus*, which is also associated with skin infections (Figure 5). In addition, *S. aureus* is one of the most common pathogenic bacteria associated with respiratory infections and food poisoning (Tong et al., 2015). Furthermore, *S*. Typhimurium was strongly inhibited by the supernatant of BL (Figure 5). This result is consistent with the finding that two *Bifidobacterium* strains, among the 14 isolates from infant stools, inhibit *S*. Typhimurium (Lievin et al., 2000). *S*. Typhimurium is one of the most harmful food poisoning bacteria and may be present in various types of foods, including meat, fruits, vegetables, and even processed foods. Although foods contaminated with *Salmonella* appear normal, they can cause severe illnesses, particularly neurological abnormalities in the brain (Chaudhuri et al., 2018). Thus, in the food industry, it is important to prevent *S*. Typhimurium contamination in food. Interestingly, the supernatant of BL did not completely inhibit the tested probiotic isolates, although it showed marginal inhibition against some probiotics, such as *B. breve* and *L. rhamnosus* (Figure 5). Based on our results, the development and processing of foods containing the supernatant of BL may be effective in preventing contamination. Therefore, the supernatant of BL could be used as a dietary supplement, as we expect postbiotic effects, and included in food and cosmetic ingredients.

## 4 CONCLUSIONS

With the increased attention to health, probiotics are widely used as dietary supplements. Therefore, identifying new probiotic strain is important. In this study, *Bifidobacterium lactis* IDCC 4301 (BL) was isolated from the feces of breast milk‐fed infants and assessed for its safety, which included the evaluation of (i) virulence genes, (ii) antibiotic resistance genes, (iii) mobile genetic elements based on whole‐genomic analysis, (iv) antibiotic susceptibility, (v) hemolysis, (vi) biogenic amine production, (vii) single‐dose acute oral toxicity, (viii) d‐lactate formation, and (ix) endogenous enzymatic activities. Based on our results, although the tetracycline resistance gene, *tetW*, was present on the chromosome of BL, we concluded that *tetW* gene transfer is not possible because no transposase adjacent to the *tetW* gene is observed and no plasmids or other extra replicons are present. Although BL is resistant to vancomycin, it is intrinsically resistant. Regarding d‐lactate formation, BL produced low levels compared to other lactic acid bacteria. Other safety data showed no biogenic amine production, acute oral toxicity, or harmful enzymatic activity. In addition to safety assessments, BL can utilize various carbohydrates and their conjugates. Furthermore, the supernatant showed antimicrobial activity against various pathogenic microorganisms. Therefore, BL can be regarded as a safe and functional probiotic ingredient in food, cosmetic, and pharmaceutical industries.

## CONFLICT OF INTEREST STATEMENT

The authors declare no competing interests.

## CONSENT FOR PUBLICATION

All authors have read and approved the final manuscript for publication.

## Supporting information


Tables S1–S6.

## Data Availability

The data are openly available in a public repository that issues datasets with DOIs.
