# Supplementary material for: Potential of Bifidobacterium lactis IDCC 4301 isolated from breast milk‐fed infant feces as a probiotic and functional ingredient
Source: Food Sci Nutr. 2023 Feb 8;11(4):1952–64. doi: 10.1002/fsn3.3230 (PMC10084967; doi:10.1002/fsn3.3230)
Supplement: Supplementary file 1 — Tables S1–S6. [file FSN3-11-1952-s001.docx]

**Supplementary material**

**Potential of *Bifidobacterium animalis* ssp. *lactis* IDCC 4301 isolated from breast milk-fed infant feces as a probiotic and functional ingredient**

O-Hyun Ban^1,3^, Won Yeong Bang^1^, Hyeon Ji Jeon^2^, Young Hoon Jung^2^, Jungwoo Yang^1^**, Dong Hyun Kim^2^*

^1^Ildong Bioscience, Pyeongtaek-si, Gyeonggi-do 17957, Republic of Korea

^2^School of Food Science and Biotechnology, Kyungpook National University, Daegu 41566, Republic of Korea

*Corresponding author: D.H. Kim

E-mail: dhkim85@gwnu.ac.kr; Tel: +82-33-640-2333

**Co-corresponding author: J. Yang; +82-031-646-3180

E-mail: yjw@ildong.com

Table S1. List of pathogenic microorganisms and their cultivation conditions used in this study for the effect of antimicrobial activity of the supernatant of *B. animalis ssp. lactis* IDCC 4301

| Type of pathogen | Microorganism | Cultivation |  |
| --- | --- | --- | --- |
| Vaginal infection | *Candida albicans* KCTC 7122 | YM medium, 25^o^C, 24 h, aerobic condition |  |
| Oral cavity | *Streptococcus mutans* KCTC 3065 | BHI medium, 37^o^C, 24 h, aerobic condition |  |
|  | *Porphyromonas gingivalis* KCTC 5352 | TSB hemin menadione medium, 37^o^C, 24 h, anaerobic condition |  |
|  | *Fusobacterium nucleatum* subsp. *Polymorphum* KCTC 2488 | BHI medium + 1% yeast extract + 0.05% L-cysteine + 1% hemin + 0.02% menadione, 37^o^C, 48 h, anaerobic condition |  |
|  | *Prevotella nigrescens* KCTC 15081 | BHI medium + 1% yeast extract + 0.05% L-cysteine + 1% hemin + 0.02% menadione, 37^o^C, 48 h, anaerobic condition |  |
| Pathogenic | *Salmonella* Typhimurium ATCC 13311 | Nutrient medium, 37^o^C, 24 h, aerobic condition |  |
|  | *Bacillus cereus* ATCC 14579 | Nutrient medium, 37^o^C, 24 h, aerobic condition |  |
|  | *Staphylococcus aureus* ATCC 25923 | TSB hemin menadione medium, 37^o^C, 24 h, aerobic condition |  |
|  | *Enterococcus faecalis* ATCC 29212 | BHI medium, 37^o^C, 24 h, aerobic condition |  |
|  | *Streptococcus pneumonia* ATCC 49619 | BHI medium, 37^o^C, 24 h, aerobic condition |  |

Table S2. List of probiotics and their cultivation conditions used in this study for the effect of antimicrobial activity of the supernatant of *B. animalis ssp. lactis* IDCC 4301

| Microorganism | Cultivation |
| --- | --- |
| *Bifidobacterium breve* | MRS + 0.05% L-cysteine medium, 37^o^C, 24 h, anaerobic condition |
| *Streptococcus thermophilus*  *Enterococcus faecium*  *Lactococcus lactis*  *Lactobacillus rhamnosus* | MRS + medium, 37^o^C, 24 h, anaerobic condition |
| *Bacillus coagulans* | MRS + medium, 45^o^C, 24 h, 200 rpm, aerobic condition |
| *Clostridium butyricum* | MRS + 0.05% L-cysteine medium, 43^o^C, 24 h, anaerobic condition |

Table S3. The detailed information of Eggnog annotation against *B. animalis ssp. lactis* IDCC 4301

| Eggnog | Description | Count | Ratio (%) |
| --- | --- | --- | --- |
| J | Translation, ribosomal structure, and biogenesis | 153 | 8.4391 |
| A | RNA processing and modification | 1 | 0.0552 |
| K | Transcription | 79 | 4.3574 |
| L | Replication, recombination, and repair | 128 | 7.0601 |
| B | Chromatin structure, and dynamics | 0 | 0.0000 |
| D | Cell cycle control, cell division, chromosome partitioning | 16 | 0.8825 |
| Y | Nuclear structure | 0 | 0.0000 |
| V | Defense mechanisms | 45 | 2.4821 |
| T | Signal transduction mechanisms | 55 | 3.0336 |
| M | Cell wall/membrane/envelope biogenesis | 92 | 5.0745 |
| N | Cell motility | 0 | 0.0000 |
| Z | Cytoskeleton | 0 | 0.0000 |
| W | Extracellular structures | 0 | 0.0000 |
| U | Intracellular trafficking, secretion, and vesicular transport | 10 | 0.5516 |
| O | Posttranslational modification, protein turnover, and chaperones | 54 | 2.9785 |
| C | Energy production and conversion | 47 | 2.5924 |
| G | Carbohydrate transport, and metabolism | 149 | 8.2184 |
| E | Amino acid transport and metabolism | 188 | 10.3696 |
| F | Nucleotide transport and metabolism | 70 | 3.8610 |
| H | Coenzyme transport and metabolism | 28 | 1.5444 |
| I | Lipid transport and metabolism | 39 | 2.1511 |
| P | Inorganic ion transport and metabolism | 80 | 4.4126 |
| Q | Secondary metabolites biosynthesis, transport, and catabolism | 3 | 0.1655 |
| R | General function prediction only | 199 | 10.9763 |
| S | Function unknown | 377 | 20.7943 |
| Total |  | 1813 | 100 |

Table S4. Analysis of transposases in *B. animalis ssp. lactis* IDCC 4301

| Contig | Gene ID | Start | End | Direction |
| --- | --- | --- | --- | --- |
| 4301_1 | 4301_1_00005 | 6,662 | 7,957 | + |
| 4301_1 | 4301_1_00113 | 133,373 | 134,287 | + |
| 4301_1 | 4301_1_00297 | 338,430 | 339,812 | - |
| 4301_1 | 4301_1_00321 | 365,292 | 366,482 | - |
| 4301_1 | 4301_1_00500 | 567,504 | 568,835 | - |
| 4301_1 | 4301_1_01009 | 1,088,739 | 1,089,818 | + |
| 4301_1 | 4301_1_01571 | 1,590,032 | 1,591,327 | - |
| 4301_1 | 4301_1_01574 | 1,595,475 | 1,596,665 | - |

Table S5. Analysis of prophage regions in *B. animalis ssp. lactis* IDCC 4301

| Region length | Contig | Start | end | GC (%) |
| --- | --- | --- | --- | --- |
| 7.7 kb | 4301_1 | 1,653,553 | 1,661,349 | 59.41 |

Table S6. Short chain fatty acids (SCFAs) in supernatant after IDCC was cultured in MRS

| SCFA | g/L |
| --- | --- |
| Lactic acid | 2.20 |
| Acetic acid | 2.53 |
| Propionic acid | - |
| Butyric acid | - |

-: not detected
